# P05.05. The effects of mindfulness training on caregivers of children with special needs: a qualitative analysis

**DOI:** 10.1186/1472-6882-12-S1-P365

**Published:** 2012-06-12

**Authors:** T Akiva, A Arel, R Benn

**Affiliations:** 1 University of Michigan, Ann Arbor, USA

## Purpose

Parents who raise children with special needs and special educators involved in teaching this vulnerable population experience high levels of stress. While evidence suggests that mindfulness training (MT) can help reduce stress levels, little is known about the paths through which mindfulness may influence caregivers' well-being and interactions. This study investigated how caregivers who have children with developmental challenges perceive the experience of MT on their well-being, family and work relations, and caregiving practices.

## Methods

Participants for the qualitative study were drawn from a sample of a larger RCT of special educators and parents of children with special needs. Study data included reliably coded responses to open-ended questions on daily session evaluations and pre-post program evaluations, and in-depth interviews conducted prior to and during the MT program. Select classroom observations occurred. Content analysis was guided by grounded theory and conducted using Dedoose mixed-methods software.

## Results

Teachers and parents provided numerous examples of improved emotional self-regulation that resulted from in-the-moment applications of mindfulness practice to highly charged situations with children. They also describe enhanced capacities to react with empathy and self-reflection to children's developmental learning difficulties. Parents reported less reactivity in relations with family members and observed improvements in family life. Teachers reported greater success in reaching difficult students as well as working with their colleagues. These changes contributed to caregiver psychological well-being, and reductions in internalization of daily stress. Several short case studies illustrate the impact of MT on the lived experiences of parents and teachers.

## Conclusion

Findings suggest that increased mindfulness in caregiving by parents and teachers working with special needs children results in greater self-awareness and psychological well-being, and improved self-regulation and strategies for interaction with children and other significant adults. MT appears to be a potent intervention for families and school personnel whose children have developmental and behavioral difficulties.

